# The Story of the Insane from Year to Year

**Published:** 1893-11-11

**Authors:** 


### THE STORY OF THE INSANE FROM YEAR TO YEAR.

(SIXTH SERIES.)

### THE STAFFORD ASYLUM AT BURNTWOOD.

At the end of the year there was in this asylum 626 patients, being a decrease of 6 on the numbers for January 1st. One hundred and sixty-nine patients were admitted, 57 were discharged recovered, 10 were discharged relieved, and 99 died. The recoveries stand at 36·77 per cent., and the deaths at 15·84; the former being below the average and the latter above it. As to the causes of insanity in those admitted, hereditary influence heads the list with 30, and drink follows with 23. Brain diseases caused death in 42 instances, and phthisis in 21. Dr. Spence explains the high death-rate by stating that many of the patients were in very bad health when admitted, and there had also been an accumulation of aged patients and of epileptic and paralysed cases. In fact, the average death-rate for 28 years is only 10·87. The committee express "their high sense of the service of Dr. Spence." The weekly cost per head is nearly 8s. 10d.

### KIRKLAND'S ASYLUM FOR THE DISTRICTS OF GLASGOW, GOVAN, AND LANARK.

This report and the accompanying statistics embrace the period between May 1st, 1892, and April 30th, 1893. On the former date the asylum contained 230 patients, and on the latter the numbers had fallen to 227. Eighty patients were admitted during the year, 37 were discharged recovered, and 21 died. The recoveries stand at 46 per cent. on the admissions, and the deaths at 9 per cent. on the average resident. The asylum was visited by typhoid fever, by measles, and by scarlet fever; and Dr. Clark points out the impossibility of satisfactory isolation of infectious cases, there being no detached hospital on the grounds of the asylum—a serious drawback certainly. A beginning has been made with the training of nurses and attendants, and 12 of the staff have passed the required examination. The Commissioners in Lunacy say that "the asylum is managed with great ability and with excellent results."

